# Plasma folate studies in HIV-positive patients at the Lagos university teaching hospital, Nigeria

**DOI:** 10.4103/2589-0557.74995

**Published:** 2010

**Authors:** Akanmu Alani, Osunkalu Vincent, Adediran Adewumi, Adeyemo Titilope, Ernest Onogu, Akinde Ralph, Coker Hab

**Affiliations:** Department of Haematology and Blood Transfusion, College of Medicine, University of Lagos, Surulere, Lagos, Nigeria; 1 Department of Morbid Anatomy, College of Medicine, University of Lagos, Surulere, Lagos, Nigeria; 2 Department of Pharmaceutical Chemistry, College of Medicine, University of Lagos, Surulere, Lagos, Nigeria

**Keywords:** Anemia, folate, HIV infection

## Abstract

**Introduction:**

In various studies globally, the prevalence of anemia in persons with HIV infection range from 10 to 20% at initial presentation, and anemia is diagnosed in 70 to 80% of these patients over the course of HIV disease. The etiology of anemia in this group of patients has not been fully established, thus a need to evaluate the role of plasma folate as a possible etiological factor.

**Objective:**

This study was set to determine plasma folate levels in newly diagnosed, treatment naïve, HIV-positive patients, and relate this to other hematological changes.

**Materials and Methods:**

A total of 200 participants were recruited for this study, of which 100 were HIV positive, treatment naive patients who were recruited at the point of registration and 100 were HIV-negative subjects (controls). 5 ml of venous blood was collected and plasma extracted for folic acid estimation by HPLC. A full blood count, CD4 and Viral load were estimated.

**Results:**

Mean ages for control and study group were 38 ± 2.3 and 32 ± 1.7 years, respectively. Mean plasma folate concentration among the study group (5.04 *μ*g/l) was significantly lower than that for the control group (15.89 *μ*g/l; *P* = 0.0002). Prevalence of anemia among the study group was 72% (144 of 200), with a mean hemoglobin (Hb) concentration of 9.5 g/dl compared with mean Hb of 13.0 g/dl among the control group (*P* = 0.002). Plasma folate correlated positively with CD4 cell count (r = 0.304, *P*<0.05) and inversely with the viral load (r = -0.566; *P*<0.05).

**Conclusion:**

Plasma folate level is a predictor of anemia in early HIV infections.

## INTRODUCTION

Folate is an essential vitamin required for vital biochemical reactions in the human body, which includes one-carbon transfer reactions, and the formation of purines and pyrimidines for DNA and RNA synthesis.[1] Lack of folate has been undoubtedly linked to a number of disorders which includes carcinogenesis and anemia among others.[1,2] These in HIV-infected individuals could be a source of additional morbidity and increased mortality.

The role of folate in carcinogenesis has been well studied in colorectal cancers.[1] Epidemiologic studies have also associated folate deficiency with cancers of the lung, esophagus, stomach, brain, cervix, pancreas, and breast, and with leukemia.[3,4] It has been proposed that because folate is required for the synthesis of deoxythymidylate from deoxyuridylate,[5] the misincorporation of uracil during DNA synthesis could be a possible mechanism for carcinogenesis in folate deficiency. Similarly, it has also been proposed that alterations in DNA methylation due to folate deficiency could contribute to carcinogenesis by modulating gene expression.[6] Folate may affect immune cell proliferation and responsiveness due to its crucial role in nucleotide synthesis.[5]

Proliferation of various cell types have also been reported in folate deficiency.[7–9] Cells lacking folate have been shown to accumulate in the S phase due to nucleotide imbalance and slow DNA synthesis; it has also been reported that such cells also have increased uracil misincorporation and DNA damage.[9,10] Documented studies have shown that when folate is added back to these folate-deficient cells, there is a reversal of the S phase accumulation, and proliferation is restored.[9,10]

In human beings, folate deficiency has been shown to reduce the proportion of circulating T lymphocytes and their proliferation in response to mitogen activation, which in turn decreases resistance to infections.[11] There is also the association of folate deficiency with faster disease progression after infection of T lymphocytes by HIV type 1.[12,13] Risk of acute lymphocytic leukemia is said to increase with low folate status.[14,15]

Anemia in HIV-infected individuals occur in 10 to 20% at presentation and are diagnosed in 70 to 80% of patients over the course of the disease,[16,17] thus making it more common than thrombocytopenia or leukopenia in patients with AIDS. The etiology of anemia in HIV infection remains largely unclear. In recent years, several attempts have been made to elucidate the mechanisms leading to HIV-associated anemia. Direct infection of erythroid progenitors have been discussed,[18] but has not been proven. Furthermore, soluble factors like HIV proteins and cytokines have been suggested to inhibit growth of hematopoietic cells in the bone marrow of HIV-infected patients. So far, no emphatic statement could be made whether these factors are directly involved in myelosuppression or mediate their effect by inhibiting growth factor synthesis.[18] Other implicated factors include changes in cytokine production with subsequent effects on hematopoiesis;[19] opportunistic infectious agents such as *Mycobacterium avium* complex and parvovirus B-19;[20,21] administration of therapeutic agents such as zidovudine,[22] ganciclovir,[23] and trimethoprim-sulfamethoxazole;[24] and myelophthisis caused by cancers such as lymphosarcoma.[23] However, in sub-Saharan Africa where a good proportion of the populace are undernourished, nutritional anemias have become a major source of concern in the investigation of anemia in this group of patients and has thus formed the focus of this study. Most of the studies on anemia in HIV infection have been focused on iron studies, with less emphasis on folate and vitamin B12 levels. Folate is rapidly depleted in diseases, especially when there is prolonged anorexia, increased metabolic rate, and high cell turnover.[25] The HIV virus is known to have a rapid turnover and can generate up to 10 billion viron in 24 hours,[26] and the implication for this is a rapid depletion of folate stores. Plasma folate level ranges from 3 to 15 *μ*g/l.[25] The human daily requirement approximates to 50 *μ*g daily in infancy, to about 100 *μ*g daily in adults.[25] The actual amount is determined by metabolic and cell turnover rate. The total body folic acid stores are 5 to 10 ml with a half-life of about 0.7 hours.[1,25] It is widely and rapidly distributed throughout the body. Large amount is stored in the liver as methylated folic acid (34%), while about 66% is bound to plasma proteins.[1,27] 20 to 90% of ingested folic acid is excreted as folinic acid in the urine within 24 hours.[1,27]

Anemia has been associated with progression to AIDS and shorter survival time for HIV-infected patients.[28] Few studies have shown that if anemia had developed, recovery from it may be associated with improved survival.[29] Understanding the association between anemia and survival is important because treatments for anemia are available.

## MATERIALS AND METHODS

One hundred HIV-positive patients, who attended the out-patient Clinic of the Lagos University Teaching Hospital, Idi-Araba, between the month of September and December, 2008, were recruited by systematic random sampling at the point of registration after obtaining verbal consent from the patients and ethical approval from the hospital ethical committee. Lagos is a southwestern state of Nigeria, noted for its multiethnic characteristics, and one of the few HIV treatment centers in Nigeria, supported by the Aids Prevention Initiative in Nigeria, a nongovernmental organization. The participants included were those confirmed positive for HIV infection by Enzyme-linked immunosorbent assay (ELISA) technique, antiretroviral naïve, within World Health Organization stage II, and with no clinical evidence of metabolic or systemic disorders. One hundred (100) HIV-negative participants were recruited by systematic random sampling from the blood donor clinic, having passed the donor criteria with a minimum hemoglobin (Hb) concentration of 12.5 g/dl.

0.5 ml of the plasma from each anticoagulated venous blood from both the patients and the control subjects were deproteinized with 1 ml of acetonitrite and centrifuged at a speed of 3 000 rpm to obtain a clear supernatant, which were subsequently injected into the High-performance liquid chromatography (HPLC) machine (by AGILENT, model 1100 series, Germany) for analyte separation and detection. Hematological investigations were done with the Sysmex automated cell counter to obtain the values of white blood cell count, Hb concentration, platelet count, and mean cell volume (MCV). CD4 cell count and viral load estimations were obtained by flow cytometry and RNA-Polymerase chain reaction, respectively. Quality control samples were run with each batch of test.

## RESULTS

As enumerated in Table 1, the mean ages of participants in the HIV study group and control were 38 ± 2.3 and 32 ± 1.7 years, respectively. Mean plasma folate levels for study group and control group were 5.04 ± 1.2 and 15.89 ± 1.8 g/l, respectively (*P* = 0.0002). Mean Hb concentration for the HIV-positive participants were 9.5 ± 2.1 g/dl compared with a mean value of 13.0 ± 1.6 g/dl in the control group (*P* = 0.002). The MCV of the study group at 85 ± 3.8 fl was significantly higher than that for the control group with mean value of 80 ± 2.9 fl (*P* = 0.04).

**Table 1:** Demographic profile with mean folate and mean hemoglobin levels

Cases	Mean age (years)		Mean folate level (μg/l)		Mean Hemoglobin level (μg/l)	
	HIV positive group	Control group	HIV positive group	Control group	HIV positive group	Control group
Male	39	32	5.05	15.93	3.92	13.5
Female	36	25	4.9	14.2	9.86	12.5
Combined mean	38	32	5.04	15.89	9.5	13.0

In Table 2, low plasma folate levels (<3.0 *μ*g/l) were demonstrated in 48% of all the HIV-positive subjects and in 52.8% of HIV-positive subjects with Hb concentration below 10 g/dl. Plasma folate levels correlated positively with CD4 cell count (r = 0.304, *P*<0.05) and inversely with the viral load count (r = −0.566; *P*<0.05) in HIV-positive participants.

**Table 2:** Relating plasma folate levels to other hematological parameters in HIV positive subjects

Parameters	Folate values μg/ml (%)		X^2^	*P* value
	< 3	> 3		
Hemoglobin (Hb) concentration g/dl				
<10	38 (52.8)	34 (47.2)	2.35	0.125
>10	10 (35.7)	18 (64.3)		
White blood cell (WBC) count				
<2 500	0 (0)	2 (100)		0.267
>2 500	48 (49)	50 (51) (Fisher exact test)		
Platelet (×10^9^)				
<100	0 (00)	6 (100)		0.027 (Fisher exact test)
>100	48(51.5)	46(48.9)		
CD4 cell count (cells/μl)				
<200	20 (58)	14 (42)	2.95	0.08
>200	26 (40)	38 (60)		
Viral load (RNA copies/ml)				
<50 000	22 (42)	30 (58)	4.08	0.04
>50 000	30 (62.5)	18 (37.5)		

## DISCUSSION

Many studies around the world have documented persistently high occurrence of anemia in HIV-infected persons, especially as disease progresses;[16,17] these have been variously linked to poor dietary intake, effect of chronic inflammation, and opportunistic infections.[20,21] However, a large percentage of asymptomatic HIV-infected persons have shown early occurrence of anemia even in the absence of attributable etiological factors.[16] Friis *et al*., in a study of HIV-positive asymptomatic subjects in Southern Brazil, reported a plasma folate deficiency in 41% of the subjects studied.[30] This study postulates that the rapid viral replication characteristic of early phase of viral infection and consequent viral burden is capable of significant reduction in folate concentration and likelihood of anemia in the asymptomatic stages of HIV infection. This could explain the significant difference in the mean values of plasma folate level of 15.89 ± 1.8 *μ*g/l among control subjects and 5.04 ± 1.2 *μ*g/l in the study population (*P* = 0.0002). This position is supported by Friis *et al*.,[30] who in a study of 1 669 subjects in Zimbabwe, recorded a significantly lower plasma folate levels among the HIV-positive study group. Therefore, HIV could be said to be a negative predictor of plasma folate probably resulting from reduced intake, impaired absorption, or increased catabolism, or a combination of these factors. The Spearman’s correlation coefficient among the study population indicated that the viral load significantly varies inversely with the plasma folate level in subjects studied (r = 0.566; *P*<0.05).

The study showed a significant association between low plasma folate levels (<3 *μ*g/l) and falling values of CD4 cell count of the subjects studied; 69.7% of these subjects with plasma folate level below 3 *μ*g/l had CD4 cell counts below 200 cells/*μ*l (*P* = 0.0004; OR, 2.92). These findings are in tandem with reports by Courtemanche *et al*.[31] who described higher CD4 apoptosis in moderate level of folate deficiency. The study also reported DNA damage and maturation arrest in the S-phase of the cell cycle which was reversible on folate repletion. Folate status is known to modulate immunocompetence and resistance to infections, and to affect cell-mediated immunity by reducing circulating T-lymphocytes and decreasing response to mitogen activation.[11] Therefore, a combination of these mechanism in a folate deficient immunocompromised host could result in a rapid deterioration, and faster progression to AIDS. The occurrence of anemia, as shown in Table 2 (Hb concentration below 10 g/dl), was demonstrated in about 52.8% of the HIV-positive population studied (*P* = 0.03); Castro *et al*.,[32] in a study of HIV-infected asymptomatic subjects in Southern Brazil, reported a prevalence of 41%. However, other studies have found no changes or even higher plasma folate levels in HIV-infected persons; these could strengthen earlier position that anemia in HIV-positive subjects is multifactorial.

## CONCLUSION

We may, from our findings, conclude that folate deficiency occurs as an early event in HIV infection, and that the introduction of folate prophylaxis may be justified as soon as HIV diagnosis is confirmed to delay disease progression and early occurrence of anemia.

## References

[1] Mason JB, Choi SW (2000). Folate and carcinogenesis: Developing a unifying hypothesis. Adv Enzyme Regul.

[2] Selhub J, Rosenberg IH (1996). Folic Acid. Present Knowledge in Nutrition.

[3] Choi SW, Mason JB (2000). Folate and carcinogenesis: An integrated scheme. J Nutr.

[4] Richard SB, Emilio M, Carl E, Mariana K, Baum F (1988). Altered folate metabolism in early HIV infect. JAMA.

[5] Ramakrishnan U (2002). Prevalence of micronutrient malnutrition worldwide. Nutr Rev.

[6] Jacob RA, Gretz DM, Taylor PC, James SJ, Pogribny IP, Miller BJ (1998). Moderate folate depletion increases plasma homocysteine and decreases lymphocyte DNA methylation in postmenopausal women. J Nutr.

[7] Koury MJ, Price JO, Hicks GG (2000). Apoptosis in megaloblastic anemia occurs during DNA synthesis by a p53-independent, nucleoside-reversible mechanism. Blood.

[8] Zhu WY, Melera PW (2001). Basal levels of metallothionein I and II expression in mouse embryo fibroblasts enhance growth in low folate through a cell cycle mediated pathway. Cell Biol Int.

[9] Huang RF, Ho YH, Lin HL, Wei JS, Liu TZ (1999). Folate deficiency induces a cell cycle-specific apoptosis in HepG2 cells. J Nutr.

[10] Koury MJ, Horne DW (1994). Apoptosis mediates and thymidine prevents erythroblast destruction in folate deficiency anemia. Proc Natl Acad Sci USA.

[11] Dhur A, Galan P, Hercberg S (1991). Folate status and the immune system. Prog Food Nutr Sci.

[12] Baum MK, Shor-Posner G, Lu Y, Rosner B, Sauberlich HE, Fletcher MA (1995). Micronutrients and HIV-l disease progression. AIDS.

[13] Boudes P, Zittoun J, Sobel A (1990). Folate, vitamin B12, and HIV infection. Lancet.

[14] Koury MJ, Park DJ, Martincic D, Home DW, Kravtsov V, Whitlock JA (1997). Folate deficiency delays the onset but increases the incidence of leukemia in Friend virus-infected mice. Blood.

[15] Skibola CF, Smith MT, Kane E, Roman E, Rollinson S, Cartwright RA (1999). Polymorphisms in the methylenetetrahydrofolate reductase gene are associated with susceptibility to acute leukemia in adults. Proc Natl Acad Sci USA.

[16] Zon LI, Arkin C, Groopman JE (1988). Haematological manifestations of the human immunodeficiency virus (HIV). Semin Hematol.

[17] Sullivan PS, Hansol DL, Chu SY, Jones JL, Ward JW (1998). Epidemiology of anaemia in human immunodeficiency virus infected persons: Results from the Multistate Adult and Adolescent Spectrum of HIV Disease Surveillance Project. Blood.

[18] Kreuzer KA, Rockstroth JK (1997). Pathogenesis and Pathophysiology of anaemia in HIV infection. Ann Hematol.

[19] Zauli G, Re MC, Visani G, Furlini G, Mazza P, Vignoli M (1992). Evidence for a human immunodeficiency virus type-l- mediated suppression of un infected hematopoietic (CD34+) cells in AIDS patients. J Infect Dis.

[20] Horsburgh CR (1991). Mycobacterium avium complex infection in the acquired immunodeficiency syndrome. N Engl J Med.

[21] Naides SJ, Howard EJ, Swack NS, True CA, Stapleton JT (1993). Parvovirus B19 infection in human immunodeficiency virus type 1-infected persons failing or intolerant to zidovudine therapy. J Infect Dis.

[22] Richman DD, Fischl MA, Grieco MH, Gottleib MS, Volderding PA, Laskin OL (1987). The toxicity of azidothymidine (AZT) in the treatment of patients with AIDS and AIDS-related complex: A double-blinded, placebo controlled trial. N Engl J Med.

[23] Faulds D, Heel RC (1990). Ganciclovir: A review of its antiviral activity, pharmacokinetic properties and therapeutic efficiency in cytomegalovirus infections. Drugs.

[24] Keisu M, Wiholm BE (1990). Trimethoprim-su1phamethoxazole-associated blood dyscrasias: Ten years’ experience of the Swedish spontaneous reporting system. J Intern Med.

[25] Rothenberg SP (1999). Increasing the dietary intake of folate: Pros and Cons. Semin Hematol.

[26] Babafemi T (2004). General HIV Medicine.

[27] Hemberg M (1999). Lymphocyte subsets as prognostic markers for cancer patients receiving immunomodulative therapy. Med Oncol.

[28] Coodley G, Girard DE (1991). Vitamins and minerals in HIV infection. J Gen Intern Med.

[29] van den Broek NR, White SA, Neilson JP (1998). The relationship between asymptomatic human immunodeficiency virus infection and the prevalence and severity of anemia in pregnant Malawian women. Am J Trop Med Hyg.

[30] Friis H, Gomo E, Koestel P, Ndhlovu P, Nyazema N, Krarup H (2001). HIV and other predictors of serum folate, serum ferritin, and hemoglobin in pregnancy: A cross-sectional study in Zimbabwe. Am J Clin Nutr.

[31] Courtemanche C, Elson-Schwab I, Mashiyama ST, Kerry N, Ames BN (2004). Folate deficiency inhibits the proliferation of primary human CD8+ T lymphocytes in vitro. J Immunol.

[32] Castro L, Goldani LZ (2009). Iron, folate and vit B12 parameters in HIV infected patient with anaemia in southern Brazil. Trop Doct.

